# Dioxygen Activation by a Bioinspired Tungsten(IV) Complex

**DOI:** 10.1021/acs.inorgchem.3c00228

**Published:** 2023-03-29

**Authors:** Miljan Z. Ćorović, Ferdinand Belaj, Nadia C. Mösch-Zanetti

**Affiliations:** Institute of Chemistry, Inorganic Chemistry, University of Graz, 8010 Graz, Austria

## Abstract

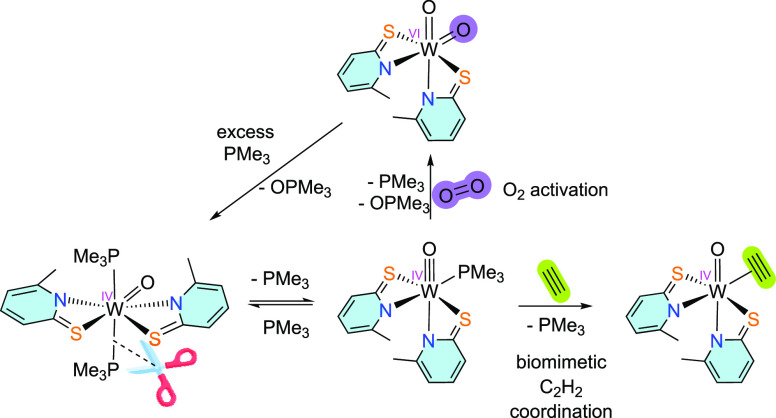

An increasing number of discovered tungstoenzymes raises interest in the biomimetic chemistry of tungsten complexes in oxidation states +IV, +V, and +VI. Bioinspired (sulfur-rich) tungsten(VI) dioxido complexes are relatively prevalent in literature. Still, their energetically demanding reduction directly correlates with a small number of known tungsten(IV) oxido complexes, whose chemistry is not well explored. In this paper, a reduction of the [WO_2_(6-MePyS)_2_] (6-MePyS = 6-methylpyridine-2-thiolate) complex with PMe_3_ to a phosphine-stabilized tungsten(IV) oxido complex [WO(6-MePyS)_2_(PMe_3_)_2_] is described. This tungsten(IV) complex partially releases one PMe_3_ ligand in solution, creating a vacant coordination site capable of activating dioxygen to form [WO_2_(6-MePyS)_2_] and OPMe_3_. Therefore, [WO_2_(6-MePyS)_2_] can be used as a catalyst for the aerobic oxidation of PMe_3_, rendering this complex a rare example of a tungsten system utilizing dioxygen in homogeneous catalysis. Additionally, the investigation of the reactivity of the tungsten(IV) oxido complex with acetylene, substrate of a tungstoenzyme acetylene hydratase (AH), revealed the formation of the tungsten(IV) acetylene adduct. Although this adduct was previously reported as an oxidation product of the tungsten(II) acetylene carbonyl complex, here it is obtained via substitution at the sulfur-rich tungsten(IV) center, mimicking the initial step of the first shell mechanism for AH as suggested by computational studies.

## Abstract 2

A tungsten(VI) dioxido complex with biomimetic pyridine-2-thiolate ligands reacts with PMe_3_ to a phosphine-stabilized tungsten(IV) compound. This reduced compound [WO(6-MePyS)_2_(PMe_3_)_2_] undergoes dissociation of one phosphine ligand in solution. The dissociation creates a vacant coordination site capable of activating dioxygen and acetylene.

## Introduction

Over the past decades, tungsten(VI) and especially molybdenum(VI) dioxido motifs have drawn much attention as models for different metalloenzymes which catalyze oxygen atom transfer (OAT) reactions.^1−5^ The major drawback of molybdenum OAT catalysts is their high tendency to form relatively inert molybdenum(V) μ-oxo dimers, which appear as a result of comproportionation of molybdenum(IV) oxido and molybdenum(VI) dioxido species.^6−8^ The dinucleation can be inhibited by increasing the steric bulk of the ligands^9^ or immobilizing the active species to a polymeric support.^10^ Analogous dimers are rare in tungsten chemistry due to the low redox potential of tungsten(VI) dioxido moieties.^11−15^ Although some of the tungsten complexes turned out as bioinorganic models and catalysts for oxygenation reactions, those reactions have a low atom economy since the presence of terminal oxidants such as peroxides is always required.^6,16^ Thus, investigating systems that utilize dioxygen or even air is highly desirable for economic and environmental reasons.^17^ Although several tungsten complexes react with dioxygen,^18−20^ no homogeneous system is reported to utilize it catalytically. In contrast, there are several examples of dioxygen activation reported for molybdenum complexes which catalyze aerobic oxidation of organic substrates^21−23^ or PMe_3_.^24−27^ The isolation of reduced complexes using a method starting from tungsten(VI) dioxido species with pyridine/pyrimidine-2-thiolate ligands and PMe_3_ was only recently reported.^14^ For this reason, there is a general lack of information on the chemistry of such reduced tungsten(IV) phosphine-stabilized species.

Besides dioxygen, much attention is also being dedicated to activating and functionalizing simple hydrocarbons.^28,29^ Hydration of acetylene with a biomimetic complex for the enzyme acetylene hydratase (AH) remains challenging.^30,31^ This tungstoenzyme contains a tungsten(IV) center coordinated by two sulfur-rich metallopterin cofactors, a cysteine residue, and an oxygen donor (water or hydroxide).^32,33^ During acetylene hydration catalysis, tungsten probably does not change its oxidation state, and according to computational studies, hydration might occur via a first shell mechanism which includes coordination of acetylene.^34−39^ Interestingly, the formation of acetylene adducts was not reported for tungsten(IV) dithiolene complexes, although these ligands are structurally most similar to the biological coordination sphere.^1^ To address this issue, we investigated the acetylene coordination to tungsten centers with various sulfur-rich ligands, however, synthetic routes always included coordination of alkynes to tungsten(II) centers followed by oxidation to the corresponding biomimetic tungsten(IV) alkyne adducts.^40,41^ Crane et al. reported coordination of acetylene to the reactive [Tp’WO(CO)I] [Tp’ = hydridotris-(3,5-dimethylpyrazolyl)borate)] complex containing both oxido and carbonyl ligands, before the crystal structure of AH was reported.^42,43^ Coordination of acetylene here occurs upon substitution of the carbonyl ligand that is simultaneously oxidized to CO_2_ with trimethylamine-*N*-oxide. Furthermore, although synthesized from tungsten(II) carbonyl precursor, [WO(C_2_H_2_)(S-Phoz)_2_] [S-Phoz = 2-(4′,4′-dimethyloxazoline-2′-yl)thiophenolate] shows photoreversible acetylene binding, indicating the possibility of acetylene coordination to tungsten(IV).^44^ Interestingly, a monophosphine molybdenum(IV) complex with 6-methylpyridine-2-thiolate ligands reacts with acetylene by substituting a phosphine ligand.^45^ This behavior resembles the binding of acetylene in AH, which was suggested to take place via substitution of the O donor ligand at the sulfur-rich tungsten(IV) center according to some computational studies.^37−39^ The analogous tungsten complex has not yet been described. Additional studies are required to better understand the coordination of acetylene to biomimetic tungsten centers and to support the suggested first shell mechanism of AH.

Inspired by the natural choice of tungsten(IV) centers in enzymes to activate different substrates, here, we describe the reduction of a tungsten(VI) dioxido complex with 6-methylpyridine-2-thiolate ligands with PMe_3,_ which leads to the formation of a phosphine-stabilized tungsten(IV) oxido compound. This reduced compound partially releases one PMe_3_ ligand in solution, creating a vacant site capable of activating dioxygen or binding acetylene. Furthermore, tungsten-catalyzed aerobic oxidation of PMe_3_ was performed with the tungsten center cycling between oxidation states +IV and +VI.

## Results and Discussion

### Preparation of [WO_2_(6-MePyS)_2_] (**1**)

**Scheme 1:**
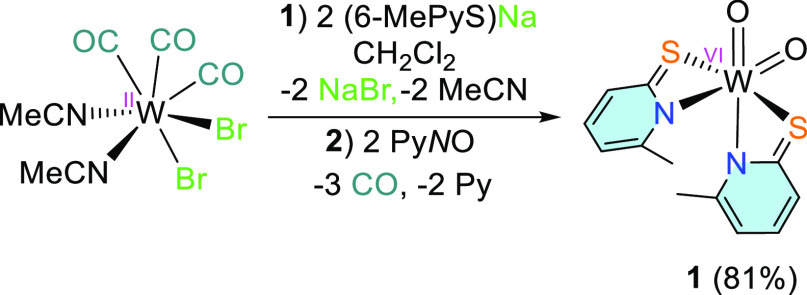
Synthesis of [WO_2_(6-MePyS)_2_] (**1**) Starting from [WBr_2_(CO)_3_(MeCN)_2_]

The reaction of [WBr_2_(CO)_3_(MeCN)_2_] with 2 equiv of sodium 6-methylpyridine-2-thiolate [Na(6-MePyS)] in CH_2_Cl_2_ led to the tricarbonyl complex [W(CO)_3_(6-MePyS)_2_].^41^ After filtration, two equiv of pyridine-*N*-oxide were directly added and the solution was stirred overnight. After adding MeCN and cooling to −25 °C, oxidized [WO_2_(6-MePyS)_2_] (**1**) precipitated and was collected by filtration in the form of a bright yellow crystalline powder in 81% yield (Scheme 1).

Complex **1** is well soluble in chlorinated solvents and benzene, moderately soluble in MeCN, and poorly soluble in aliphatic hydrocarbons and ether. In benzene, compound **1** is stable for at least 24 h, even when purged with dioxygen. However, it is not stable in a DMSO solution where the bidentate ligands dissociate and oxidize to the respective disulfide (6-MePyS)_2_ accompanied by the reduction of DMSO to dimethyl sulfide and the formation of unidentified tungsten oxides (WO_3_)_*n*_, a behavior previously observed for [WO_2_(PymS)_2_] (PymS = pyrimidine-2-thiolate).^14^ The complex exists as a single isomer in solution, as confirmed by ^1^H and ^13^C NMR spectroscopy. The IR stretching characteristic for W=O bonds were detected at 907 and 949 cm^–1^, comparable to similar W^VI^O_2_ complexes.^14^

**Figure 1:**
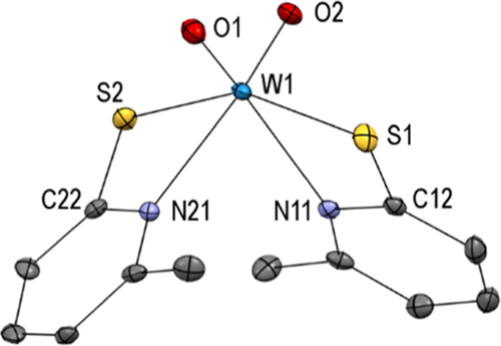
Molecular structure (50% probability ellipsoids) of complex [WO_2_(6-MePyS)_2_] (**1**) showing the atomic numbering scheme. H atoms are omitted for clarity.

Single crystals suitable for X-ray diffraction analysis were obtained from CH_2_Cl_2_/MeCN at −37 °C. The molecular structure (Figure 1) reveals a distorted octahedral geometry of the tungsten center coordinated by two sulfur atoms oriented *trans* to each other [S1–W1–S2 152.68(3)°] and two nitrogen atoms oriented *trans* to the oxido ligand [O1–W1–N11 154.01(11)°; O2–W1–N21 154.11(11)°], which is isotypic with the known [MoO_2_(6-MePyS)_2_] analogue.^46^ However, the W=O bonds are with 1.717(3) and 1.727(2) Å, respectively, slightly elongated in the tungsten analogue **1**, as previously observed for comparable tungsten and molybdenum systems.^14,46,47^ Presumably, due to packing forces, the torsion angles around the C–S bonds of the two ligands are significantly different [e.g., W1–S1–C12–N11–11.5(2)° vs W1–S2–C22–N21 1.6(2)°]. The 6-methyl group in the ligand leads to slightly longer W–N bonds [W1–N11 2.376(3) Å; W1–N21 2.326(3) Å] when compared to previously published complexes with similar ligands [e.g., in [WO_2_(PyS)_2_] (PyS = pyridine-2-thiolate) W1–N11 2.300(2) Å; W1–N21 2.287(2) Å].^14^

### Reduction Studies with PMe_3_

**Scheme 2:**
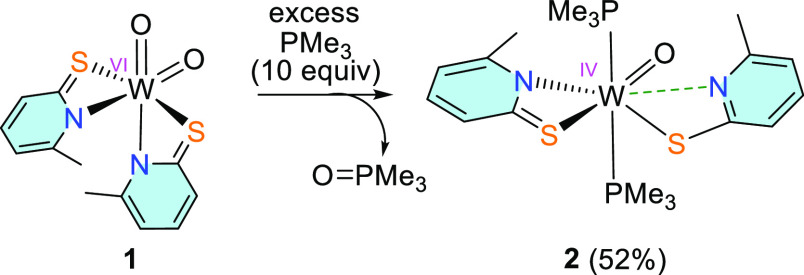
Formation of [WO(6-MePyS)_2_(PMe_3_)_2_] (**2**) under Reduction of **1** with Excess PMe_3_

Reaction of **1** with excess of PMe_3_ in CH_2_Cl_2_ and stirring overnight results in a dark green reaction mixture. The addition of *n*-heptane and cooling to −25 °C for 4 weeks led to the gradual decrease of the solution color intensity alongside the formation of orange crystals of the tungsten(IV) compound [WO(6-MePyS)_2_(PMe_3_)_2_] (**2**) (Scheme 2).

The IR spectrum of **2** shows one strong band at 928 cm^–1^ indicative of ν(W=O), which is in accordance with the literature data of similar complexes.^14,48^ Furthermore, the purity of the sample was confirmed by elemental analysis.

**Figure 2:**
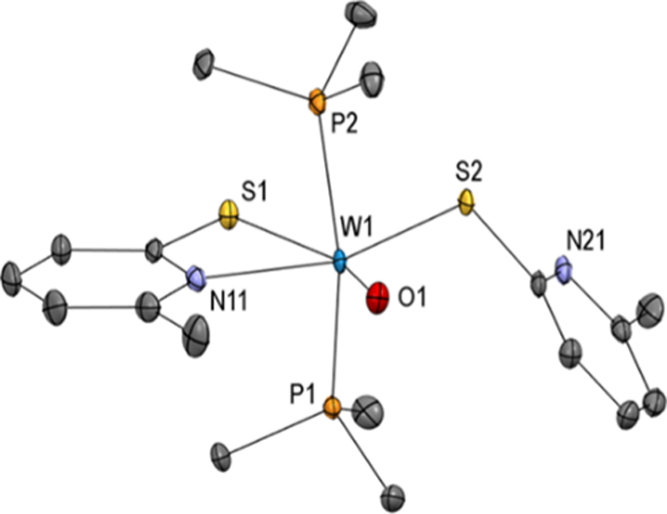
Molecular structure (50% probability ellipsoids) of [WO(6-MePyS)_2_(PMe_3_)_2_] (**2**) showing one bidentate and one monodentate 6-methylpyridine thiolate ligand. H atoms and solvent molecules are omitted for clarity.

The solid-state structure of **2** was determined by single-crystal X-ray diffraction analysis (Figure 2). Single crystals suitable for X-ray diffraction analysis were obtained from CH_2_Cl_2_/*n*-heptane at −37 °C from a solution of **2** containing 10 equiv of PMe_3_. The analysis reveals a distorted octahedral environment of the W atom consisting of two PMe_3_ ligands in *trans* position [P1–W1–P2 angle ≈ 162.08(5)°], one oxido group, one bidentate ligand, and one monodentate 6-methylpyridine-2-thiolate ligand, the latter being coordinated via its sulfur atom. The O ligand is found trans to one of the sulfur atoms [O1–W1–S1 160.44(13)°]. For this reason, the W1–S1 bond [2.6464(13) Å] is significantly longer compared to W1–S2 [2.3907(12) Å]. Furthermore, the orientations of the ligands are in contrast with the dioxido starting material where the nitrogen atoms are found trans to the oxido groups. The diversity of the coordinative situation with the ligands represents an important feature for the found reactivities described below and allows the formation of intermediates that are otherwise not accessible.

The molecular structure of **2** should be compared to that of [WO(PyS)_2_(PMe_3_)_2_] lacking the methyl groups in the ligands (Table 1). The lower steric demand in [WO(PyS)_2_(PMe_3_)_2_] allows both ligands to coordinate in a bidentate fashion in contrast to **2**. Except for one nitrogen tungsten bond/distance, the two structures exhibit very similar bond lengths. The more electron-donating ligands and the lower coordination number in 2 than in [WO(PyS)_2_(PMe_3_)_2_] leads to slightly shorter bonds.

**Table 1:** Selected Bond Lengths in [WO(PyS)_2_(PMe_3_)_2_] and **2**

bond lengths [Å]	[WO(PyS)_2_(PMe_3_)_2_]^14^	**2**
W–O1	1.745(2)	1.711(4)
W–S1	2.6675(8)	2.6463(13)
W–S2	2.6668(10)	2.3907(12)
W–N1	2.205(3)	2.206(4)
W–N2	2.195(3)	
W–P1	2.4760(8)	2.5159(13)
W–P2	2.4883(9)	2.4859(14)
S1–C12	1.726(4)	1.732(5)
S2–C22	1.719(3)	1.787(5)

**Scheme 3:**
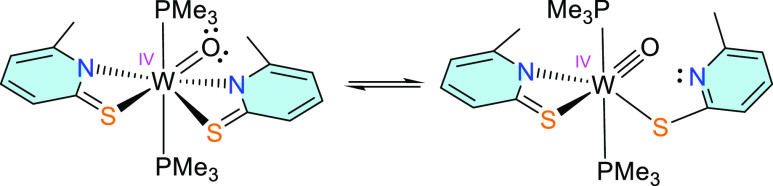
Dynamic Behavior of **2** in Solution Caused by De-/Coordination of One Nitrogen Donor of the Ligand

Characterization of **2** by NMR spectroscopy revealed a complex solution behavior. ^1^H NMR spectroscopy of a solution of in situ formed **2** in CDCl_3_, which contains an excess of PMe_3_ (10 equiv), confirms the formation of a species with two equiv of coordinated PMe_3_ molecules. Next to the resonance for uncoordinated PMe_3_, all other signals are assignable to the symmetric hepta-coordinated [WO(6-MePyS)_2_(PMe_3_)_2_] with two PMe_3_ ligands (Figure S6). The *trans* orientation of two phosphines is demonstrated by the virtual triplet arising in the aliphatic region in the ^1^H NMR spectrum (δ 1.32 ppm, CDCl_3_) and a singlet flanked by ^183^W satellites in the ^31^P{^1^H} NMR spectrum (δ −22.72 ppm, CDCl_3_). The formation of a bisphosphine species is analogous to tungsten complexes containing pyridine/pyrimidine thiolate ligands^14^ but in contrast to the monophosphine adduct observed for the Mo compound bearing the identical 6-MePyS ligands.^46^ The unsymmetric coordination of the two 6-MePyS ligands found in the solid state of **2** is in contrast to its solution behavior where only one type of ligand was detected by ^1^H NMR spectroscopy pointing toward a dynamic behavior as presented in Scheme 3.

**Figure 3:**
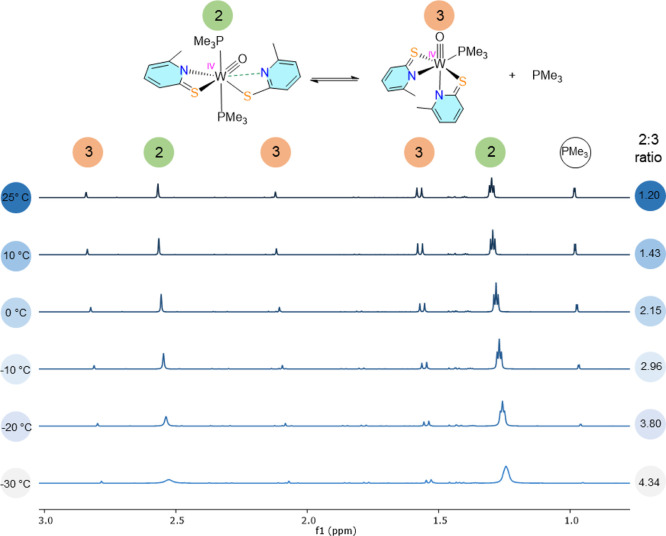
Variable temperature ^1^H NMR spectroscopy of **2** in CD_2_Cl_2_ solution showing a thermodynamic equilibrium in the aliphatic region. The aromatic region is omitted for clarity.

NMR spectroscopy of a pure sample of **2** in CD_2_Cl_2_ in the absence of excess PMe_3_ revealed the formation of a second tungsten compound in solution. The ^1^H NMR spectrum at 25 °C shows resonances for **2** as well as for [WO(6-MePyS)_2_(PMe_3_)] (**3**) in which only one phosphine ligand is coordinated together with one uncoordinated PMe_3_ molecule (Figure 3). Coordination of only one PMe_3_ in **3** renders the two bidentate ligands asymmetric showing two sets of ligand resonances, while a doublet at 1.58 ppm belongs to one coordinated PMe_3_. Additionally, the ^31^P{^1^H} NMR signal deriving from the coordinated phosphine in **3** appears at −17.50 ppm, which is upfield shifted by 20 ppm in comparison to [MoO(6-MePyS)_2_(PMe_3_)],^46^ due to the lower π basicity of molybdenum.

To get further insights into this behavior, variable temperature NMR spectroscopy of single crystals of **2** in CD_2_Cl_2_ was performed. Lowering the temperature from 25 to −30 °C shifts the ratio of **2** versus **3** from 1.20:1 to 4.34:1 (Figure 3), which goes along with a color change from brown to deep green. This demonstrates that, in solution, compound **2** is in equilibrium with **3** under the decoordination of one phosphine. The presence of an equilibrium in solution is further supported by the fact that the ^1^H NMR spectrum of **2** with excess of phosphine reveals no resonances of **3** as described above. Thus, at a lower temperature, more **2** is formed, which is in accordance with the entropy. By flushing a solution of **2** with N_2_ at room temperature to remove the released low boiling PMe_3,_ the color changes to brown by shifting the equilibrium to **3**. However, the VT ^1^H NMR spectra give no evidence for the occurrence of an isomeric species of **2** with unsymmetric ligands, as found by X-ray diffraction analysis. The resonances of **2** are found to broaden upon lowering the temperature, but splitting is not observed, pointing toward a very fast equilibrium between **2** and its unsymmetric isomeric form (Scheme 3).

Dynamic behavior of the ligand was also observed in the related [WO(PymS)_2_(PMe_3_)_2_]. In this example, pyrimidine-2-thiolate ligands can interchange the donor N atom upon rotation about the W–S bond which also causes broadening of the signals in ^1^H NMR spectrum.^14^

While the equilibrium between **2** and **3** can be influenced by flushing with N_2_, a complete shift toward **3** was not possible, but decomposition was observed. Thus, compound **3** seems unstable in the absence of excess phosphine, contrasting the molybdenum example.^46^

**Figure 4:**
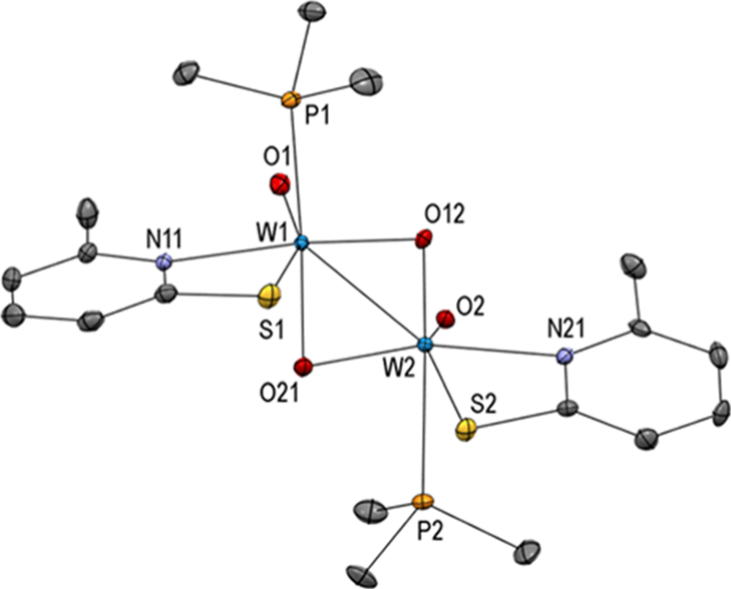
Molecular structures (50% probability ellipsoids) of complex **4**. H atoms and solvent molecules are omitted for clarity.

To get insights into the formation of other possible reduction products of **1**, the latter was treated with one equiv of phosphine. On one occasion, a small number of red single crystals formed. X-ray diffraction analysis revealed the dinuclear tungsten(V) compound [WO_2_(6-MePyS)(PMe_3_)]_2_ (**4**) as displayed in Figure 4. The two tungsten(V) centers are bridged by two μ-oxido groups forming a W_2_O_2_ ring and are additionally coordinated by one terminal oxido, one bidentate 6-MePyS ligand, and one trimethylphosphine ligand leading to a distorted octahedral surrounding. The two oxido ligands are almost eclipsed to each other [e.g., O1–W1–W2–O2 0.78(16)°]. The analogous dimer is described to crystallize upon treating [MoO_2_(6-MePyS)_2_] with sub-stoichiometric amounts of PMe_3_.^46^

Although the isolation of compound **4** in larger quantities proved futile, its formation delivers important information concerning the reduction process of complex **1**. Most probably, PMe_3_ coordinates to the tungsten in [WO_2_(6-MePyS)_2_] in an initial step as similar behavior was observed in related tungsten alkyne complexes.^49^ In the absence of further equivalents of phosphine, compound **4** is formed, and only with additional PMe_3_ molecules OAT occurs, forming **2**. The initial coordination of PMe_3_ might be enabled by the flexibility of the 6-MePyS ligand, as observed in compound **2** (Scheme 3). Initial phosphine coordination probably enhances the following OAT step by weakening the W=O bond.

### Dioxygen Activation

Since complex **3** can reversibly bind PMe_3_, we tested the possibility of activation of dioxygen as a more challenging substrate. Reactions of monophosphine molybdenum(IV) oxido species with dioxygen lead either to oxido η^2^–peroxido complexes [MoO(O_2_)L_2_] or dioxido complexes [MoO_2_L_2_] depending on the steric demand of the bidentate ligands L.^24,26,50^ Similar studies with tungsten compounds have not been investigated. Compound **2** was dissolved in benzene, and the solution was flushed with O_2_. After 90 min under an O_2_ atmosphere, the solvent was removed. After suspending the solids in CH_2_Cl_2_ and filtration through Celite, the filtrate was overlaid with *n*-heptane, and yellow crystalline material was isolated from the solution and identified as dioxido complex **1** in 80% yield (eq 1).
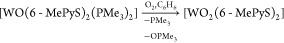
1

Inspired by this result, the catalytic properties of complex **1** for the oxidation of PMe_3_ to OPMe_3_ with O_2_ were investigated (Table 2). Therefore, a reaction flask with **1** (5 mol %) was filled with O_2_, loaded with the benzene solution of PMe_3_, closed, and stirred for 24 h at rt. The conversion was calculated by integrating the signals of PMe_3_ and OPMe_3_ in ^1^H and ^31^P{^1^H} NMR spectra. An additional experiment was carried out with [WO_2_(PyS)_2_]^14^ to elucidate the effect of the methyl substituent in [WO_2_(6-MePyS)_2_]. Furthermore, the catalytic experiment with 1 mol % of **1** was performed under identical conditions as previously published for molybdenum catalysts with iminophenolate ligands to compare different systems and to test the catalytic activity under lower catalyst loading.^25,26^ Comparison to molybdenum is included due to the lack of comparable tungsten systems. Results are presented in Table 2. Detailed experimental conditions can be found in the Supporting Information.

**Table 2:**
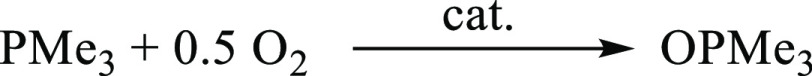
Results of Catalytic Aerobic Oxidation of PMe_3_

entry	complex^a^	cat. load. (mol %)	*V*_flask_ (mL)	conversion (%)	ref
1	[WO_2_(6-MePyS)_2_]	5	50	43	
2	[WO_2_(PyS)_2_]	5	50	17	
3	[WO_2_(6-MePyS)_2_]	1	100	58^i^	
4	[MoO_2_L^a^_2_]	1	100	19	(26)
5	[MoO_2_L^b^_2_]	1	100	65	(25)

a L^a^ = 2,4-di-*tert*-butyl-6-[(*tert*-butylimino)methyl]phenolate; L^b^ = 2,4-di-*tert*-butyl-6-{[(3-methoxypropyl) imino]methyl}phenolate. ^i^Reaction is not selective. Oxygenation products: OPMe_3_ (main product), OP(OMe)Me_2_,_,_ and P(OMe)Me_2_.

Blank experiments without complex revealed no more than 5% of PMe_3_ oxidation. Complex **1** shows a significantly higher conversion than the analogous one without an additional methyl group [WO_2_(PyS)_2_]. This suggests that prior to the coordination of dioxygen to the reduced tungsten(IV) oxido compound, one molecule of phosphine must dissociate to create an empty coordination site. As described above, compound **2** is in equilibrium with a species with only one coordinated phosphine which is not observed with [WO(PyS)_2_(PMe_3_)_2_].^14^ The higher concentration of the monophosphine intermediate in the catalytic reaction with **1** likely explains the higher conversion obtained.

Under reaction conditions identical to those previously published for molybdenum compounds^25,26^ (entry 3), non-selective oxidation occurs with catalyst **1,** probably due to higher O_2_ content (100 mL Schlenk flask vs 50 mL Schlenk flask). Three products were identified: OPMe_3_ (68% of total conversion), methoxydimethylphosphine oxide [OPOMe(Me)_2_] (27% of total conversion), and methoxydimethylphosphine [POMe(Me)_2_] (5% of total conversion) (see the Supporting Information). The formation of byproducts was confirmed by comparison with literature data.^25,51^

**Scheme 4:**
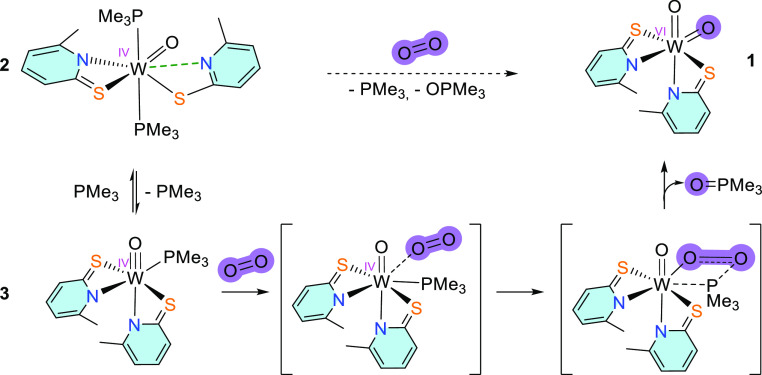
Proposed Mechanism of Tungsten(IV) Complex Oxidation with O_2_

It is noteworthy that η^1^–superoxido or η^2^–peroxido complexes have not been observed via NMR spectroscopy during the stoichiometric or catalytic reactions. Nevertheless, it could be envisioned that a species such as [WO(O_2_)(6-MePyS)_2_] is formed quickly in an initial step which is reacting quickly with PMe_3_. Thus, we wondered whether reactivity with other oxygen acceptors is occurring. For this reason, 10 equiv of cyclooctene were added to a solution of **2** in benzene, which was purged with O_2_ and allowed to stir under an O_2_ atmosphere for 24 h. However, no reaction occurred, as confirmed by GC–MS analysis. Therefore, it seems conceivable that the superoxido and peroxido tungsten intermediates are not formed, but rather a mechanism presented in Scheme 4 for the oxidation of the tungsten(IV) complex takes place.

The suggested mechanism includes a tungsten(IV) transition state stabilized with an η^1^-dioxygen moiety via σ-type interactions. Further reaction of tungsten(IV)-activated O_2_ with coordinated PMe_3_ allows the isolation of **1** followed by thermodynamically favorable P=O bond formation. A similar mechanism was suggested for the reaction between [WO(PyS)_2_(PMe_3_)_2_] and DMSO, where one PMe_3_ decoordinates and the resulting monophosphine complex with a vacant site polarizes the DMSO leading to the formation of [WO_2_(PyS)_2_] and DMS.^14^ Another possibility is that a free PMe_3_ could act as the nucleophile for the terminal oxygen atom of the η^1^-bound dioxygen. However, an intramolecular reaction seems more likely due to the lack of reactivity with cyclooctene. Mechanisms involving unsaturated phosphine-free [WOL_2_] species are not considered since the formation of similar compounds was reported to be highly endergonic.^14^

**Scheme 5:**
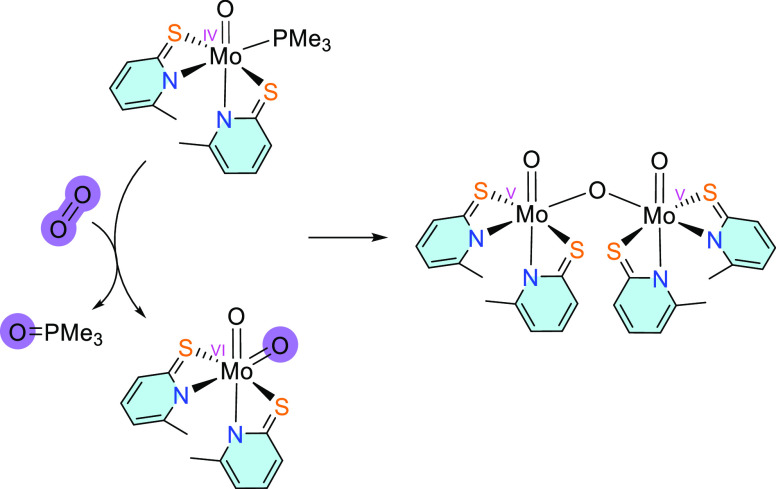
Reaction of the Molybdenum(IV) Complex Leads to the Isolation of the Molybdenum(V) Dinuclear Complex

Since there are no known tungsten complexes which react with dioxygen to form η^2^–peroxido species, it is not clear whether their formation is prevented due to the inherent nature of the metal center or the coordination environment. For this reason, we investigated the reactivity of the molybdenum analogue [MoO(6-MePyS)_2_(PMe_3_)] with 1 or 2 equiv of O_2_ in CD_2_Cl_2_. However, in both cases, IR, EI–MS, and UV vis data revealed the formation of [Mo_2_O_3_(6-MePyS)_4_], the comproportionation product between the molybdenum(IV) monooxido and molybdenum(VI) dioxido complex (Scheme 5).^46,52^

As previously described, such a comproportionation reaction does not occur with W complexes bearing similar ligands.^14^ However, isolation of [Mo_2_O_3_(6-MePyS)_4_] gives evidence that oxidation to the molybdenum(VI) dioxido intermediate occurred, demonstrating that both M(IV) complexes (M = Mo, W) react similarly with O_2_.

The oxidation of the molybdenum(IV) center with dioxygen is followed by the decoordination of the phosphine ligand. The steric situation seems to play an essential role as it was previously shown that β-ketiminato complexes of the type [MoO(ON)_2_(PMe_3_)] (ON = β-ketiminato ligands) react with O_2_ to either [MoO(O_2_)(ON)_2_] when bulkier ON ligands are used, or [MoO_2_(ON)_2_] when ligands where sterically less demanding as also found in this work.^50^ Also, the formation of stable η^2^–peroxido complexes has been observed with monophosphine molybdenum(IV) complexes with sterically encumbered iminophenolate ligands.^24−27^ Presumably, the η^1^–dioxygen complex is initially formed, but the interaction between phosphine and the terminal oxygen is sterically prevented, which results in oxidative addition of the dioxygen and formation of the η^2^–peroxido species.

### Acetylene Coordination

**Scheme 6:**
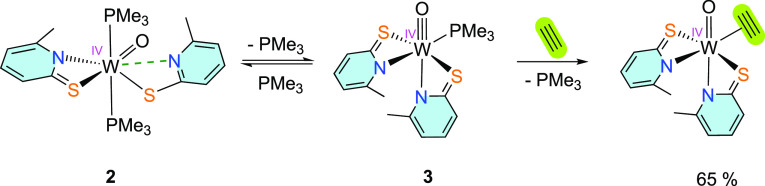
Coordination of Acetylene to a Sulfur-Rich Tungsten(IV) Complex

Since the molybdenum(IV) analogue of **3** has previously been reported to react with acetylene by substitution of PMe_3_ to yield the corresponding molybdenum(IV) acetylene adduct,^45^ the reactivity of the reduced W complex toward acetylene was investigated. In situ generation of tungsten(IV) was carried out starting from **1** and 10 equiv of PMe_3_ in benzene. After complete reduction (16 h), the green solution was flushed with N_2_ for 30 min and then with acetylene for 45 min. After stirring the reaction mixture under an acetylene atmosphere overnight, the brown solution turned into a yellow suspension with a dark precipitate, most likely polyacetylene. After filtration and solvent removal, the ^1^H NMR spectrum of the product in CD_2_Cl_2_ (yellow solution) revealed the formation of [WO(C_2_H_2_)(6-MePyS)_2_] (Scheme 6) as the major product accompanied by minor impurities, which probably derived from the reactivity of acetylene with PMe_3_ as already observed when reacting the respective molybdenum species.^45^ After filtration and precipitation from CH_2_Cl_2_/*n*-heptane, the product could be isolated in 65% yield. Still, the purity is lower compared to the one recently reported by our group via the established method [oxidation of the tungsten(II) acetylene carbonyl adduct with pyridine-*N*-oxide].^41^ Nevertheless, this represents the first example of irreversible acetylene binding by substitution to a sulfur-rich tungsten(IV) center and is in accordance with the first shell mechanism suggested for AH. Coordination occurs via the substitution of PMe_3_, mimicking the substitution of a coordinated water molecule by acetylene in the first shell mechanism suggested by Himo and co-workers.^37^

## Conclusions

Inspired by oxotransferase enzymes, we designed a reactive in situ formed monophosphine tungsten(IV) oxido complex with 6-methylpyridine-2-thiolate ligands to investigate dioxygen activation. The reaction of the unusual tungsten(IV) species with dioxygen led to the respective tungsten(VI) dioxido complex. Since the reaction of the oxidized complex with PMe_3_ leads to the tungsten(IV) reduced species, it was possible to extend the studies toward tungsten-catalyzed aerobic oxidation of PMe_3_. We observed that the methyl group in the ancillary ligand increases the catalytic activity by facilitating the formation of the reactive monophosphine species, which is not observable in the case of the analogous complex without the methyl group in position 6 of the ligand. A direct comparison of complex **1** with previously published molybdenum catalysts for oxidation of PMe_3_ revealed similar catalytic potential but an unselective reactivity. For tungsten-catalyzed reactions, we suggest the η^1^–dioxygen tungsten(IV) species as a transition state, which rapidly reacts with coordinated PMe_3_ to recover the starting tungsten(VI) dioxido complex. These results provide new insights into the often-overlooked tungsten OAT chemistry and show that dioxygen can be utilized as a terminal oxidant in these reactions. Isolation of a phosphine-stabilized tungsten(IV) oxido compound is possible only because of the dynamic behavior of 6-MePyS ligands used in this research. The existence of complex [WO(6-MePyS)_2_(PMe_3_)] (**3**), formed in an equilibrium with **2** and PMe_3_, also allowed acetylene to bind to the tungsten center in the physiological oxidation state +IV by substituting the coordinated trimethylphosphine. This reaction represents the first example of a coordination of acetylene via substitution at the sulfur-rich tungsten system which is relevant to the mechanism of acetylene hydratase suggested by some computational studies.^37^

## Supporting Information Available

Full crystallographic data, NMR spectra, and experimental procedures (PDF)
