# Supplementary material for: Dioxygen Activation by a Bioinspired Tungsten(IV) Complex
Source: Inorg Chem. 2023 Mar 29;62(14):5669–76. doi: 10.1021/acs.inorgchem.3c00228 (PMC10091480; doi:10.1021/acs.inorgchem.3c00228)
Supplement: Supplementary file 1 — ic3c00228_si_001.pdf [file ic3c00228_si_001.pdf]

Supporting Information

Dioxygen Activation by a Bioinspired Tungsten(IV) Complex

Miljan Z. Ćorović,[a] Ferdinand Belaj,[a] Nadia C. Mösch-Zanetti[, a]*

[a]Institute of Chemistry, Inorganic Chemistry, University of Graz, 8010 Graz, Austria

* Corresponding author. Tel.: +43 (0)316 380 – 5286

E-mail address: nadia.moesch@uni-graz.at

Table of Contents

1 General Considerations S1
2 Catalytic Experiments S2
3 Syntheses S3
4 Crystal Structure Determination S5
5 NMR Spectra S11
6 References S18

1 General Considerations

All experiments were carried out under inert atmosphere employing standard Schlenk and glovebox techniques unless otherwise stated. All chemicals were purchased from commercial sources and except for pyridine-*N*-oxide all were used without further purification. Pyridine-*N*-oxide was recrystallized from Et_2O and sublimed at 55°C prior to use. All solvents were purified by a Pure Solv Solvent Purification System and stored over activated molecular sieves (3 Å). NMR spectra were recorded using a Bruker Avance III and Bruker Avance NEO 500 MHz spectrometer. ^1H NMR spectra were recorded at 300.13 MHz for room temperature or at 500.23 MHz for low temperature measurements and referenced to residual protons of the NMR solvents. ^{13}C NMR spectra were obtained at 75.48 MHz for room temperature or at 125.80 MHz for low temperature measurements and spectra were referenced to the deuterated solvent peak. $^{31}P\{^1H\}$ spectra were recorded at 121.49 MHz, with 85% H_3PO_4 as an external reference. The chemical shifts δ are given in ppm. The multiplicity of peaks is denoted as broad singlet (bs), singlet (s), doublet (d), triplet (t), quadruplet (q), multiplet (m), and virtual triplet (vt). Coupling constants J are given in Hertz. Mass spectrometry measurements using electron impact ionization (EI-MS) have been performed with an Agilent 5973 MSD with a push rod for direct sample measurement. IR spectra were recorded in the solid-state at a resolution of 2 cm^{-1} on a Bruker ALPHA-P Diamant ATR-FTIR. Elemental analyses (C, H, N, S) were carried out by the Department of Inorganic Chemistry at the Graz University of Technology (Heraeus Vario Elementar automatic analyzer). Each experiment was independently performed at least three times. $[WBr_2(CO)_3(MeCN)_2]$,[1] $[MoO_2(6\text{-}MePyS)_2]$, $[MoO_2(PyS)_2]$, $[WO_2(PyS)_2]$, $[Mo_2O_3(6\text{-}MePyS)_4]$, $[MoO(6\text{-}MePyS)_2(PMe_3)]$ and Na(6-MePyS) (PyS= pyridine-2-thiolate, 6-MePyS = 6-methylpyridine-2-thiolate) were prepared according to literature.[2–4] The disulfide $(6\text{-}MePyS)_2$ was synthesized as previously reported.[5]

2 Catalytic Experiments

The conversions in catalytic reactions were determined by integrating resonances of the substrate and product in ^{1}H and ^{31}P NMR spectra. All experiments were performed at least three times. In each experiment, the complex was placed in a Schlenk flask, which was first evacuated and then charged with O_2. Dry benzene-d_6 and PMe_3 were added through the septum and the reaction mixture was left to stir under the O_2 atmosphere at rt for 24h.

Experiments with 5 mol % of the catalyst: Complex (24.1 µmol, 5 mol % versus PMe_3), 50 mL Schlenk flask, dry benzene-d_6 (1.5 mL), and PMe_3 (50 µL).

Experiments with 1 mol % of the catalyst: Complex (14 µmol, 1 mol % versus PMe_3), 100 mL Schlenk flask, dry benzene-d_6 (2 mL), and PMe_3 (145 µL).

Byproducts:

$OP(OMe)Me_2$: ^{1}H NMR (300 MHz, C_6D_6): δ 3.29 (d, 3H, OMe), 0.99 (d, 6H, Me);

$^{31}P\{^{1}H\}$ NMR (121 MHz, C_6D_6) δ 49.93 ppm.

$P(OMe)Me_2$: ^{1}H NMR (300 MHz, C_6D_6): δ 3.21 (d, 3H, OMe), 1.07 (d, 6H, Me);

$^{31}P\{^{1}H\}$ NMR (121 MHz, C_6D_6) δ 122.21 ppm.

3 Syntheses

[WO_2(6-MePyS)$_2$] (1). Ligand salt Na(6-MePyS) (2.05 equiv) was added portionwise to a stirred solution of [$WBr_2(CO)_3(MeCN)_2$] (1.500 g, 2.94 mmol, 1.0 equiv) in 20 mL of CH_2Cl_2. After 45 min, the suspension was filtered through Celite and a solution of pyridine-*N*-oxide (0.560 g, 5.88 mmol, 2.0 equiv) in 10 mL of CH_2Cl_2 was added to the filtrate. The reaction mixture was stirred for 15h with a bubbler attached. The volume of the reaction mixture was then reduced to 30 mL, 15 mL of CH_3CN was added and the dark yellow crystalline powder was isolated upon slow solvent evaporation and removal of excess liquid by cannulation. The product was washed with cold CH_3CN and ether and dried in vacuo. Colorless single crystals suitable for X-ray diffraction analysis were obtained from solvent CH_2Cl_2/MeCN mixtures at -37 °C.

Yield: 1.105 g (81%). ^{1}H NMR (300 MHz, $CDCl_3$): δ 7.53 (t, J = 7.8 Hz, 2H, pyH-*p*), 6.96 (dd, J = 14.1, 7.8 Hz, 4H, pyH-*m*), 2.67 (s, 6H, CH_3) ppm. ^{13}C NMR (75 MHz, $CDCl_3$) δ 165.29 (s, 2C, pyC-*o*), 158.04 (s, 2C, pyC-*o*), 139.70 (s, 2C, pyC-*p*), 123.68 (s, 2C, pyC-*m*), 121.35 (s, 2C, pyC-*m*), 24.55 (s, 2C, CH_3) ppm. IR (cm^{-1}): 949 (s, W=O), 907 (s,W=O).). EI-MS (70eV) *m/z*: $[M]^+$ 464.1. Anal. Calcd for $C_{12}H_{12}N_2O_2S_2W$: C, 31.05; H, 2.61; N, 6.03; S, 13.81. Found: C, 31.13; H, 2.47; N, 6.09; S, 13.51.

[WO(6-MePyS)$_2$(PMe_3)$_2$] (2). A solution of PMe_3 (446 µL, 4.30 mmol, 10 equiv) in 3 mL of CH_2Cl_2 was added to **1** (200 mg, 0.43 mmol, 1 equiv) dissolved in 6 mL of CH_2Cl_2. After stirring overnight in a closed flask, the solution was overlaid with n-heptane and cooled to -37 °C. After four weeks, dark orange crystals were isolated from a deep green solution, washed with cold n-pentane, and dried under a vacuum.

Yield: 134 mg (52 %) ^{1}H NMR (300 MHz, $CDCl_3$) δ 7.05 (t, J = 7.6 Hz, 2H, pyH-*p*), 6.92 (d, J = 7.8 Hz, 2H, pyH-*m*), 6.78 (d, J = 7.4 Hz, 2H, pyH-*m*), 2.57 (s, 6H, CH_3), 1.32 (t, $^2J_{HP}$ = 4.1

Hz, 18H) ppm. ^{31}P NMR (121 MHz, $CDCl_3$) δ - 22.72 ppm. ^{13}C NMR (75 MHz, $CDCl_3$, pyC-*o* obsucred) δ 157.00 (s, 2C, pyC-*o*), 134.31 (s, 2C, pyC-*p*), 124.29 (s, 2C, pyC-*m*), 118.19 (s, 2C, pyC-*m*), 24.57 (s, 2C, CH_3), 15.36 (t, J = 14.3 Hz, 6C, PCH_3) ppm. IR (cm^{-1}): 928 (s,W=O). EI-MS (70eV) *m/z*: $[M - 2\ PMe_3]^+$ 448.2. Anal. Calcd for $C_{18}H_{30}N_2OP_2S_2W$: C, 36.01; H, 5.04; N, 4.67; S, 10.68. Found: C, 35.91; H, 4.75; N, 4.47; S, 10.30.

Upon dissolving $[WO(6\text{-}MePyS)_2(PMe_3)_2]$ in CD_2Cl_2, NMR spectra reveal the presence of **2** and **3** existing in equilibrium at rt:

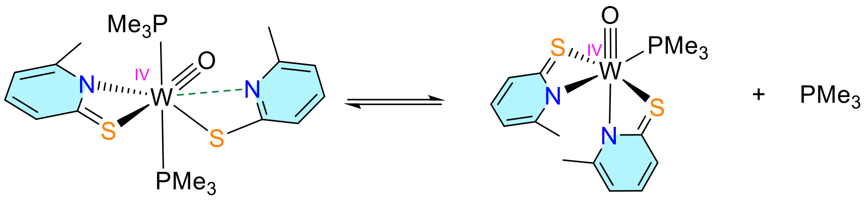

2 **3**

Scheme 1. The behavior of **2** in solution

[WO(6-MePyS)$_2$(PMe$_3$)$_2$] (**2**): ^{1}H NMR (300 MHz, Methylene Chloride-*d*$_2$) δ 7.09 (t, J = 7.7 Hz, 2H, pyH-*p*), 6.93-6.79 (m, 4H, pyH), 2.58 (s, 6H, CH_3), 1.31 (t, $^2J_{HP}$ = 4.1 Hz, 18H) ppm. ^{31}P NMR (121 MHz, CD_2Cl_2) δ - 22.95 ppm.

[WO(6-MePyS)$_2$(PMe$_3$)] (**3**): ^{1}H NMR (300 MHz, Methylene Chloride-*d*$_2$) δ 7.52 (t, J = 7.8 Hz, 1H, pyH-*p*), 6.93-6.79 (m, 3H, pyH), 6.59 (t, J = 7.7 Hz, 1H), 6.46 (d, J = 7.9 Hz, 1H, pyH-*m*), 2.85 (s, 3H), 2.13 (s, 3H), 1.58 (d, J = 9.1 Hz, 9H) ppm. ^{31}P NMR (121 MHz, CD_2Cl_2) δ - 17.50 ppm.

4 Crystal Structure Determination

Crystal Structure Determination – General. All single crystal measurements were performed on a Bruker APEX-II CCD diffractometer at 100 K using Mo K_α radiation with a wavelength of 0.71073Å from an Incoatec microfocus sealed tube equipped with a multilayer monochromator. Absorption corrections were made semi-empirically from equivalents. The structures were solved by direct methods (SHELXS-97)[6] and refined by full-matrix least-squares techniques against F^2 (SHELXL-2014/6)[7]. A weighting scheme of $w = 1/[\sigma^2(F_o^2)+(aP)^2+bP]$ where $P = (F_o^2+2F_c^2)/3$ was used. The non-hydrogen atoms were refined with anisotropic displacement parameters without any constraints.

Crystal data, data collection parameters, and structure refinement details are given in Table **S1**. Further refinement information, structure and bonding parameters, SHELXL.res, and .hkl files are given in the deposited CIF file which is available free of charge from The Cambridge Crystallographic Data Centre (CCDC 2232095-2232097).

Crystallographic Data

Table S1. Crystallographic data and structure refinement for complexes **1**, **2** and **4**.

**Crystal data**	$[WO_2(6\text{-MePyS})_2]$ (**1**)	$[WO(6\text{-MePyS-}S,N)$ $(6\text{-MePyS-}S)(PMe_3)_2]$ (**2**)	$[WO_2(6\text{-MePyS})(PMe_3)]_2\cdot$ CH_2Cl_2 (**4**)
CIF data code	MIC100B	MIC103	MIC103B
Empirical formula	$C_{12}H_{12}N_2O_2S_2W$	$C_{18}H_{30}N_2OP_2S_2W$	$C_{18}H_{30}N_2O_4P_2S_2W_2\cdot CH_2Cl_2$
Formula weight	464.21	600.35	917.12
Crystal description	block, yellow	block, orange	block, red
Crystal size [mm]	0.19 x 0.12 x 0.05	0.32 x 0.28 x 0.13	0.18 x 0.15 x 0.08
Temperature	100 K	100 K	100 K
Crystal system	Triclinic	Orthorhombic	Orthorhombic
Space group	P -1	$P\ 2_1\ 2_1\ 2_1$	P b c a
a	7.9004(4)Å	11.4487(6)Å	12.3893(5)Å
b	7.9101(4)Å	11.9817(7)Å	18.2165(8)Å
c	12.7895(7)Å	16.9156(9)Å	25.5828(12)Å
α	74.095(2)°		
β	74.826(3)°		
γ	65.137(2)°		
Volume	687.51(6)$Å^3$	2320.4(2)$Å^3$	5773.8(4)$Å^3$
Z	2	4	8
Calc. Density	2.242 mg/m^3	1.719 mg/m^3	2.110 mg/m^3
F (000)	440	1184	3488
Linear absorption coefficient μ	8.702 mm^{-1}	5.306 mm^{-1}	8.431mm^{-1}
Max. and min. transmission	1.000 and 0.850	0.746 and 0.253	0.746 and 0.312
Unit cell determination	2.89° < θ < 29.55°	2.74° < θ < 30.00°	2.89° < θ < 29.97
Reflections used	7844	9502	9532
**Data collection**			
Θ range for data collection	2.88 to 30.00°	2.08 to 30.00°	1.59 to 30.00°
Reflections collected/ unique	25098 / 3992	72049 / 6744	331840 / 8415
Significant unique reflections	3696 with I > 2σ(I)	6415 with I > 2σ(I)	7405 with I > 2σ(I)
R(int), R(sigma)	0.0880, 0.0563	0.0686, 0.0396	0.1112, 0.0366
Completeness to θ_{max}	99.7%	100%	100.0%
**Refinement**			
Data/ parameters/ restraints	3992 / 178 / 0	6744 / 253 / 0	8415 / 317 / 0
Goodness-of-fit on F^2	1.047	1.093	1.097
Final R indices [I > 2σ(I)]	R1 = 0.0269, wR2 = 0.0571	R1 = 0.0306, wR2 = 0.0731	R1 = 0.0260, wR2 = 0.0527
R indices (all data)	R1 = 0.0305, wR2 = 0.0583	R1 = 0.0328, wR2 = 0.0739	R1 = 0.0322, wR2 = 0.0550
Weighting scheme param. a, b	0.0115, 0.1622	0.0404, 1.6718	0.0184, 2.2532
Largest Δ/σ in last cycle	0.001	0.002	0.003
Largest diff. peak and hole	1.993, -1.888e/$Å^3$	1.616, -1.755e/Å	1.693 and -1.732e/$Å^3$
**CCDC no.**	2232095	2232096	2232097

.

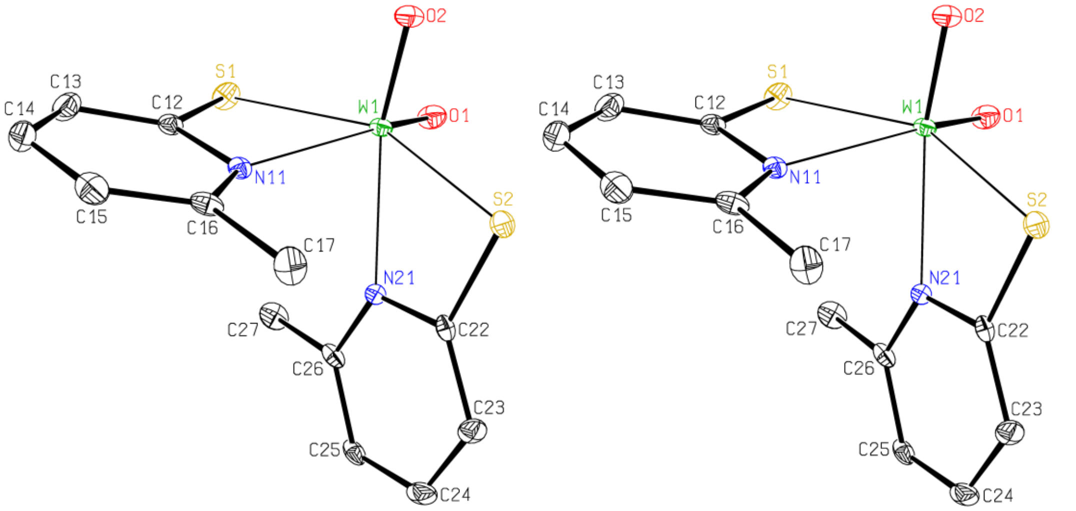

Figure S1. Stereoscopic ORTEP[8] plot of **1** showing the atomic numbering scheme. The probability ellipsoids are drawn at the 50% probability level. The H atoms were omitted for clarity.

Table S2. Selected bond lengths [Å] and angles [°] for **1**.

**Selected bond lengths [Å]**		**Angles [°]**	
W1-O1	1.717(3)	O1-W1-O2	105.92(12)
W1-O2	1.727(2)	O1-W1-N11	154.01(11)
W1-N11	2.376(3)	O2-W1-N21	154.11(11)
W1-N21	2.326(3)	N11-W1-N21	79.98(10)
W1-S1	2.4217(9)	S1-W1-S2	152.68(3)
W1-S2	2.4311(8)	C12-S1-W1	85.30(11)
S1-C12	1.760(4)	C12-N11-C16	118.8(3)
S2-C22	1.753(3)	C12-N11-W1	97.1(2)
**Torsion angles [°]**		C16-N11-W1	142.8(2)
W1-N11-C12-S1	11.8(2)	C22-S2-W1	85.07(11)
W1-S1-C12-N11	-11.5(2)	C22-N21-C26	119.7(3)
W1-N21-C22-S2	-1.7(2)	C22-N21-W1	99.4(2)
W1-S2-C22-N21	1.6(2)	C26-N21-W1	140.8(2)

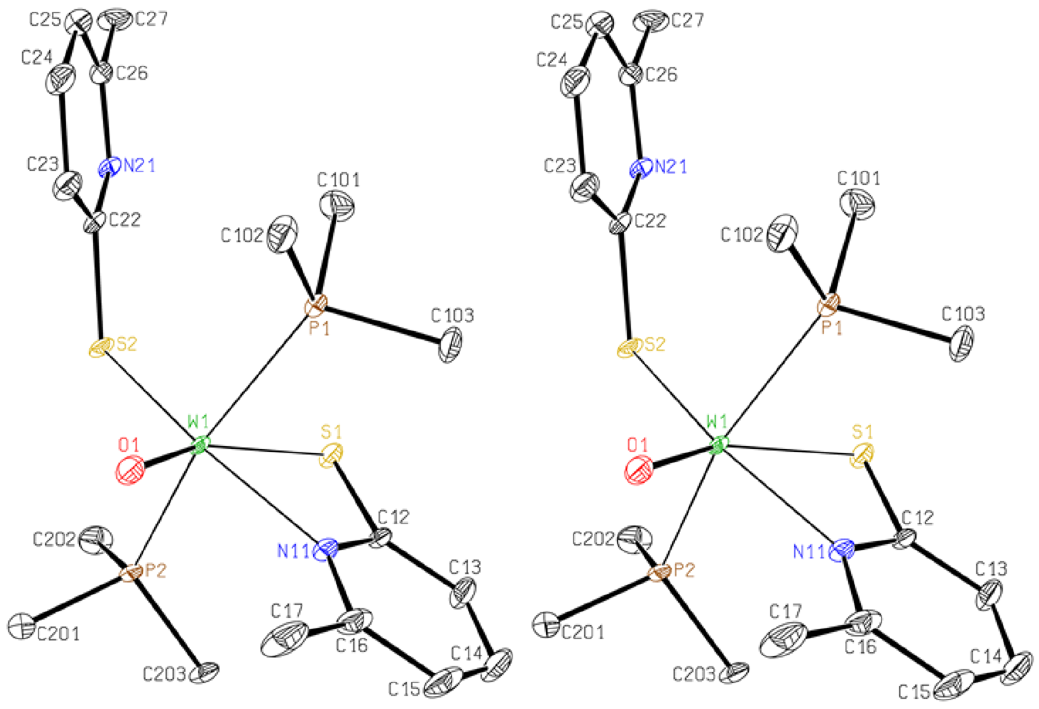

Figure S2. Stereoscopic ORTEP[8] plot of **2** showing the atomic numbering scheme. The probability ellipsoids are drawn at the 50% probability level. The H atoms were omitted for clarity.

Table S3. Selected bond lengths [Å] and angles [°] for **2**.

**Selected bond lengths [Å]**		**Angles [°]**	
W1-O1	1.711(4)	O1-W1-S1	160.44(13)
W1-N11	2.206(4)	N11-W1-S2	154.95(12)
W1-S1	2.6463(13)	P1-W1-P2	162.08(5)
W1-S2	2.3907(12)	C12-S1-W1	79.26(18)
W1-P1	2.5159(13)	C12-N11-C16	120.2(5)
W1-P2	2.4859(14)	C12-N11-W1	105.3(3)
S1-C12	1.732(5)	C16-N11-W1	134.5(4)
S2-C22	1.787(5)	C22-S2-W1	115.03(18)
**Torsion angles [°]**			
O1-W1-P1-C102	-15.8(2)		
O1-W1-P2-C201	13.0(2)		
N11-W1-P1-C103	3.31(19)		
N11-W1-P2-C203	-8.5(3)		
P2-W1-S2-C22	173.31(15)		
W1-S1-C12-N11	-0.2(3)		
W1-S1-C12-C13	178.2(5)		
W1-S2-C22-N21	132.4(3)		
W1-S2-C22-C23	-51.5(5)		

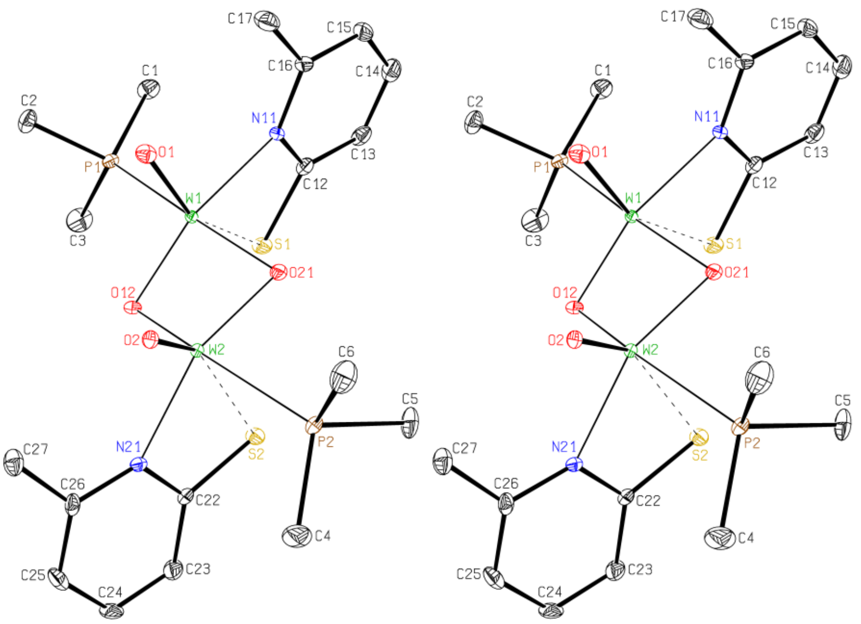

Figure S3. Stereoscopic ORTEP[8] plot of **4** showing the atomic numbering scheme. The probability ellipsoids are drawn at the 50% probability level. The H atoms were omitted for clarity.

Table S4. Selected bond lengths [Å] and angles [°] for **4**.

**Selected bond lengths [Å]**		**Angles [°]**	
W1-O1	1.726(2)	W1-O12-W2	82.18(8)
W1-O12	1.941(2)	W2-O21-W1	82.40(8)
W1-O21	1.979(2)	O12-W1-O21	93.75(9)
W1-N11	2.220(2)	O21-W2-O12	93.50(9)
W1-P1	2.5568(8)	O12-W1-N11	153.08(9)
W1-S1	2.6408(8)	O21-W1-P1	163.07(7)
S1-C12	1.733(3)	O1-W1-S1	153.98(7)
P1-C2	1.812(3)	C12-S1-W1	79.36(11)
P1-C3	1.817(3)	C16-N11-C12	120.5(3)
P1-C1	1.821(3)	C16-N11-W1	135.1(2)
W2-O2	1.721(2)	C12-N11-W1	104.42(19)
W2-O21	1.941(2)	O21-W2-N21	155.08(9)
W2-O12	1.987(2)	O12-W2-P2	162.81(6)
W2-N21	2.226(3)	O2-W2-S2	149.96(7)
W2-P2	2.5517(8)	C22-S2-W2	79.70(11)
W2-S2	2.6404(8)	C26-N21-C22	119.9(3)
S2-C22	1.725(3)	C26-N21-W2	135.8(2)
P2-C5	1.808(3)	C22-N21-W2	104.20(19)
P2-C4	1.809(3)		
P2-C6	1.815(4)		

Torsion angles [°]	
O1-W1-W2-O2	0.78(16)
S1-W1-W2-S2	-7.90(3)
W1-O12-W2-O21	-21.62(9)
O12-W2-O21-W1	21.18(8)
W2-O21-W1-O12	-21.71(8)
O21-W1-O12-W2	21.19(8)
W1-O12-W2-N21	-176.70(8)
W2-O12-W1-P1	-175.78(8)
W1-O21-W2-P2	-175.42(5)
W2-O21-W1-N11	-174.73(8)

5 NMR Spectra

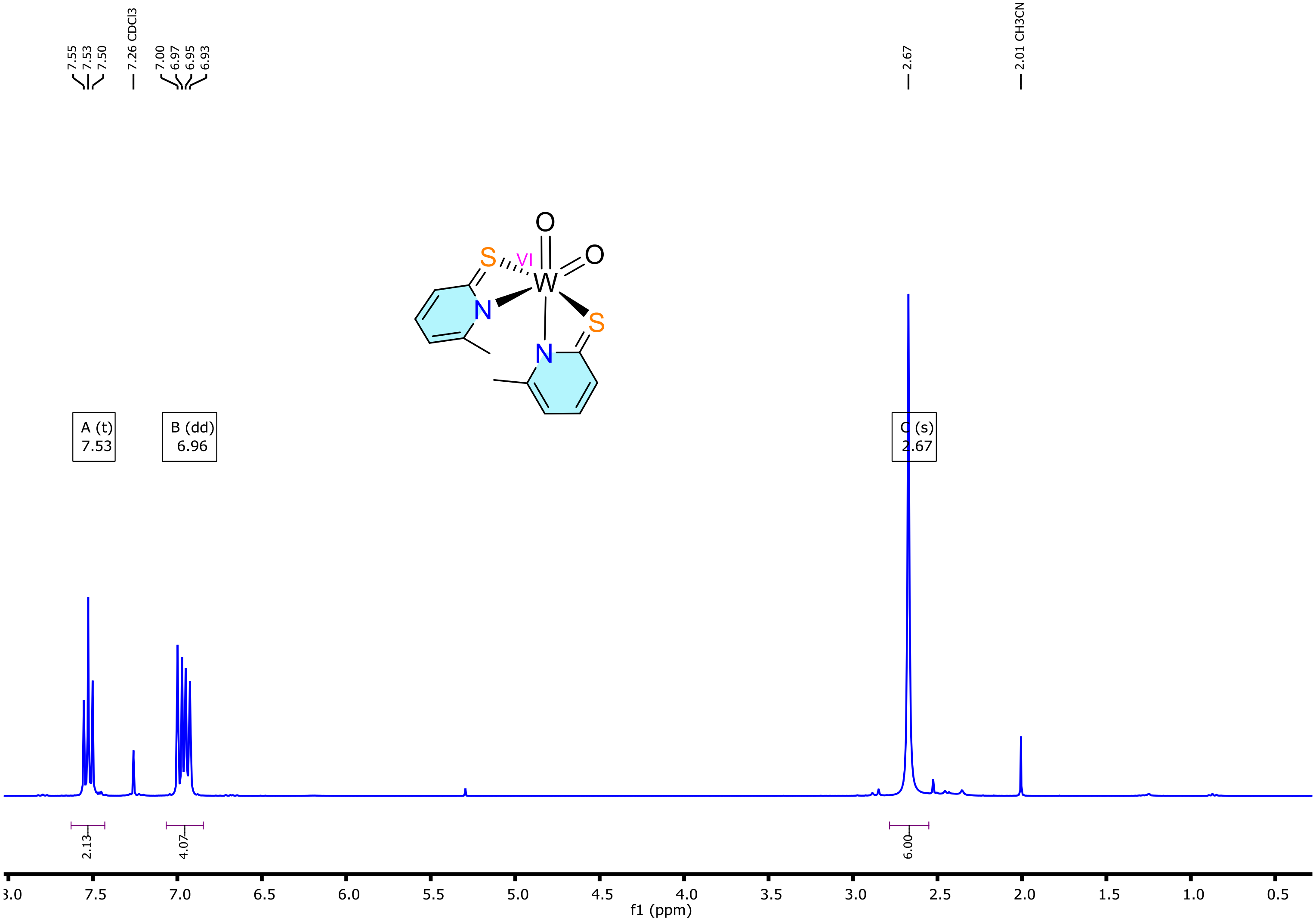

Figure S4. ^{1}H NMR spectrum of $[WO_2(6\text{-}MePyS)_2]$ (**1**) in $CDCl_3$.

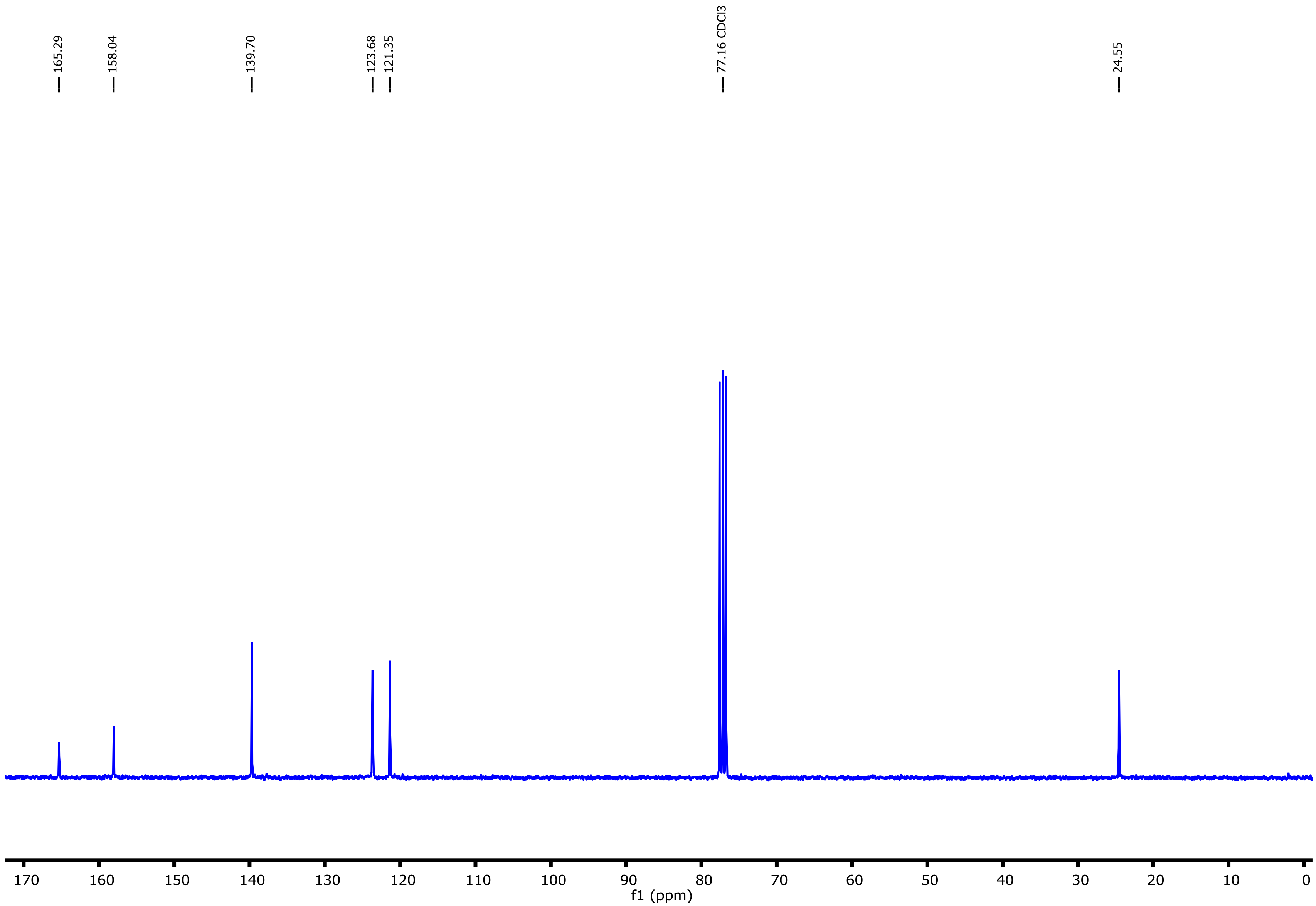

Figure S5. ^{13}C NMR spectrum of $[WO_2(6\text{-MePyS})_2]$ (**1**) in $CDCl_3$.

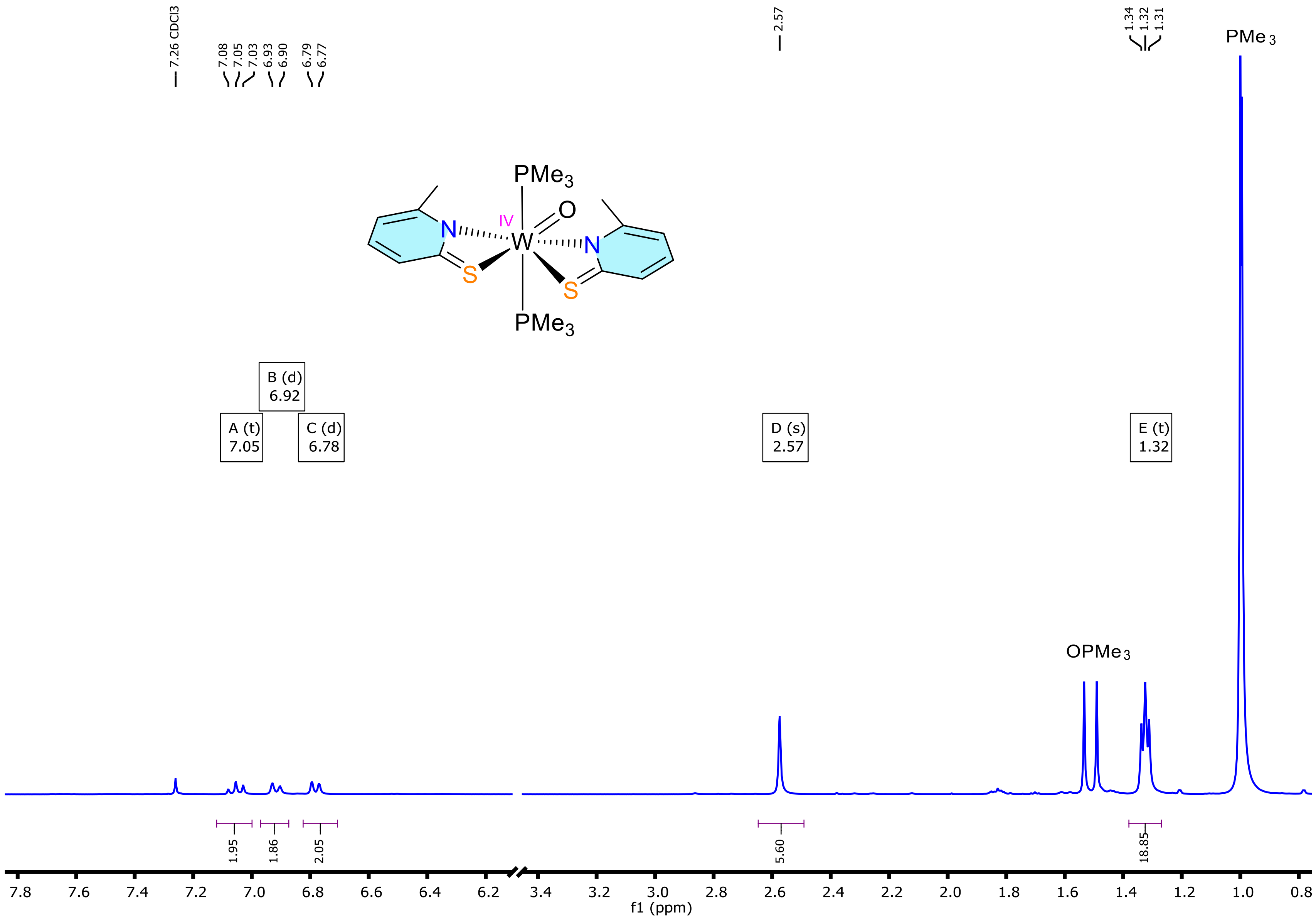

Figure S6. *In situ* ^{1}H NMR spectrum of [WO(6-MePyS)$_2$(PMe$_3$)$_2$] (**2**) in $CDCl_3$, with excess PMe$_3$ and OPMe$_3$.

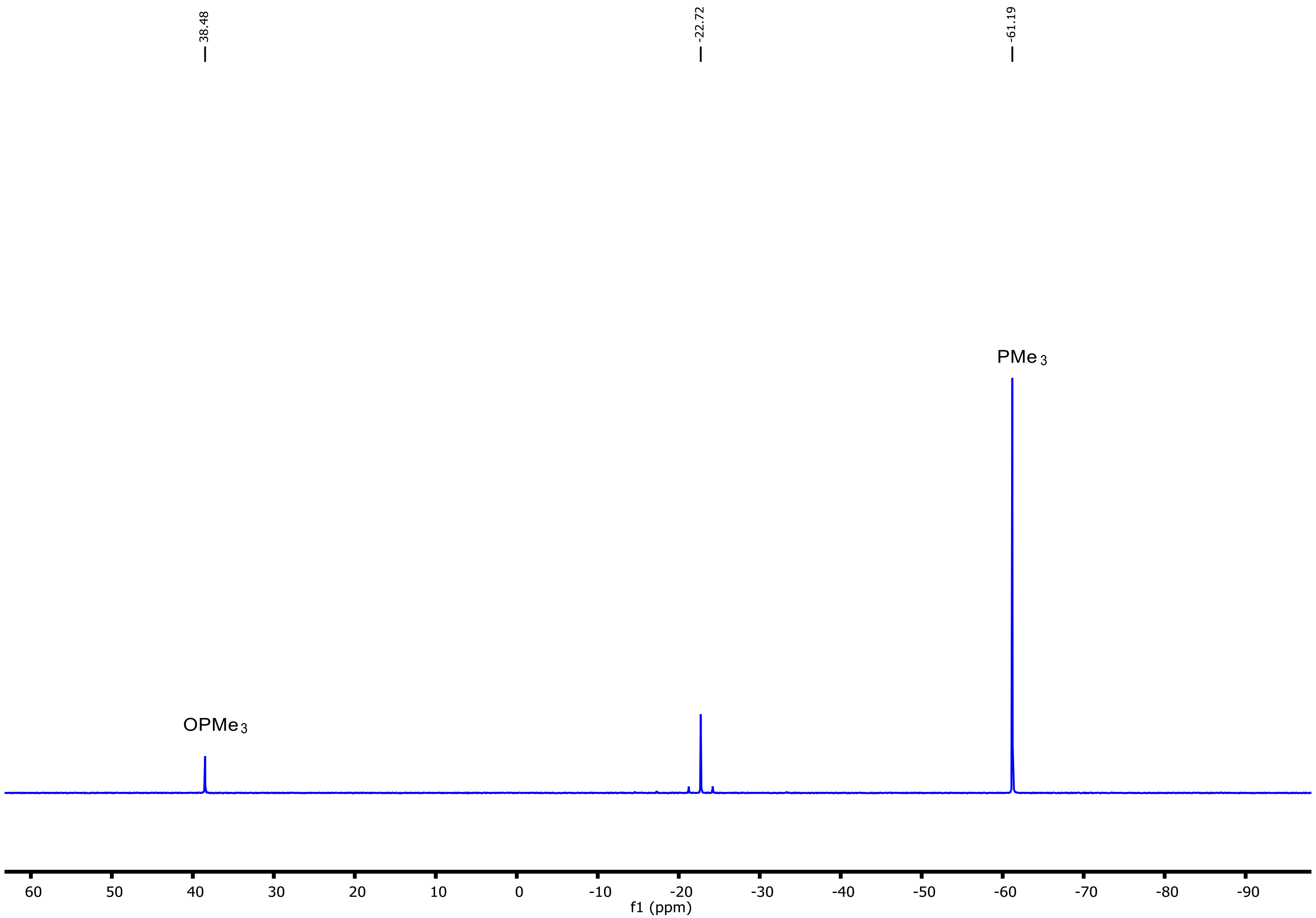

Figure S7. *In situ* ^{31}P NMR spectrum of [WO(6-MePyS)$_2$(PMe$_3$)$_2$] (**2**) in $CDCl_3$. with excess PMe_3 and $OPMe_3$.

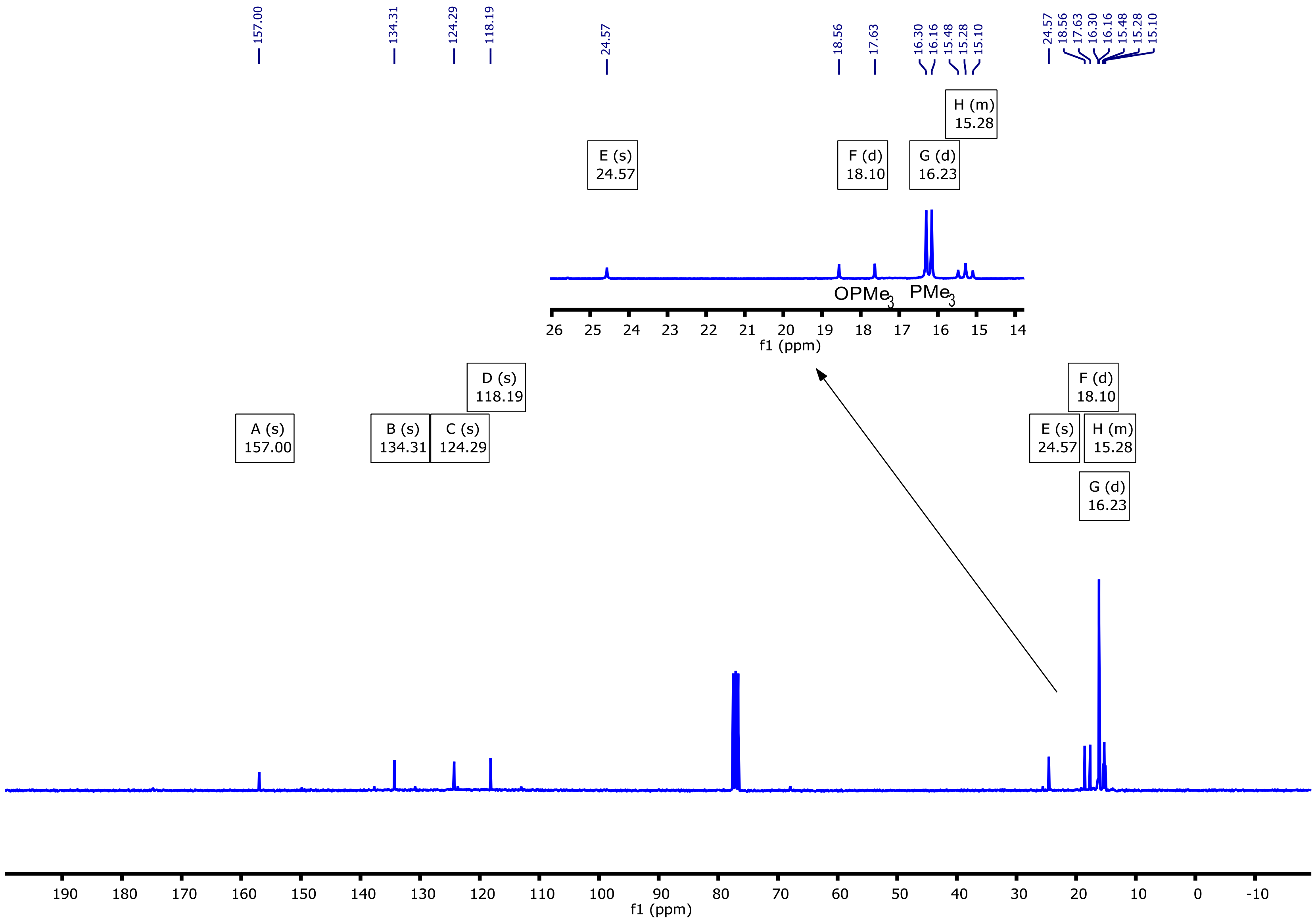

Figure S8. *In situ* ^{13}C NMR spectrum of [WO(6-MePyS)$_2$(PMe$_3$)$_2$] (**2**) in $CDCl_3$. with excess PMe_3 and $OPMe_3$.

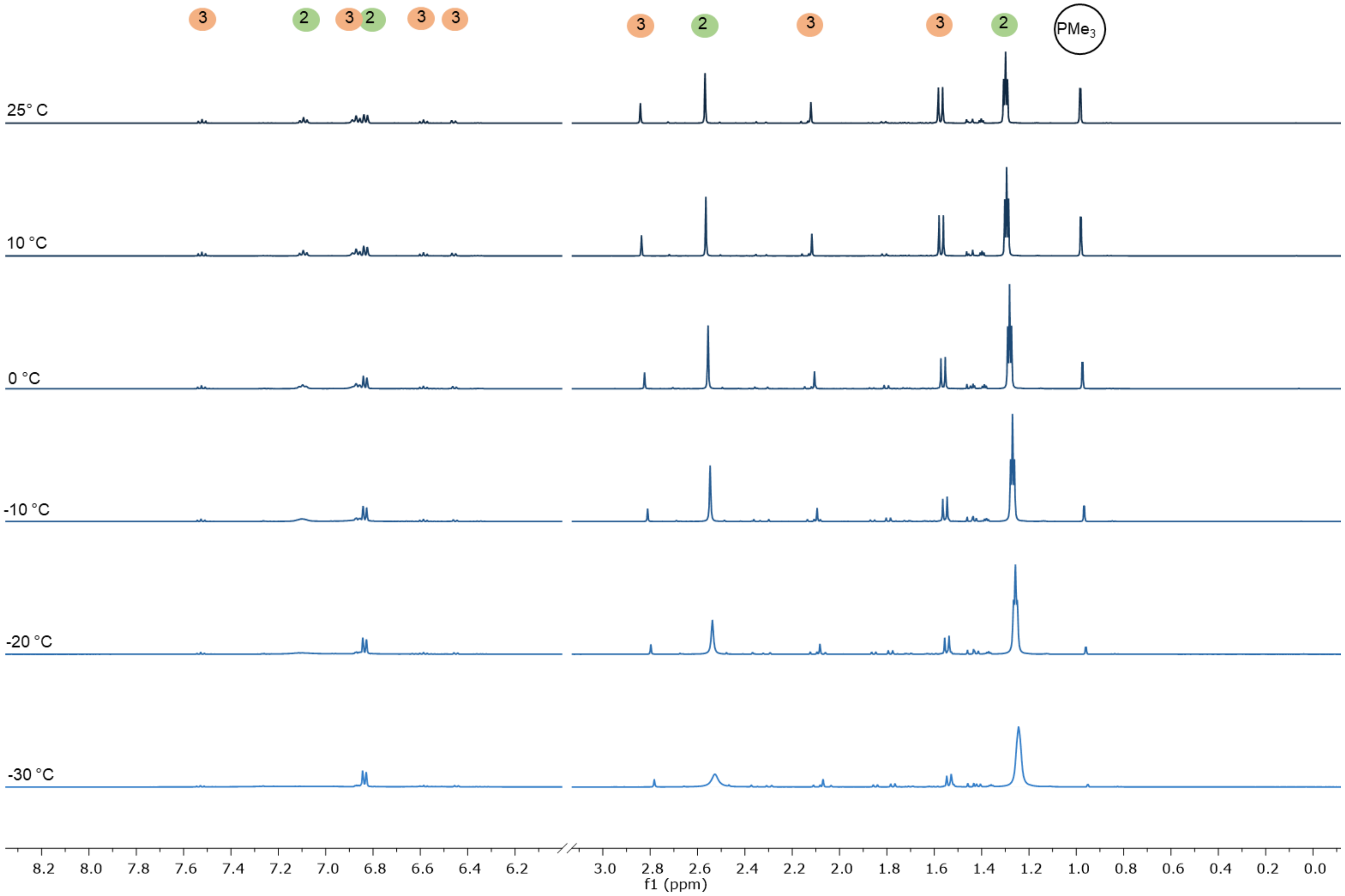

Figure S9. VT NMR of complex **2** in CD_2Cl_2.

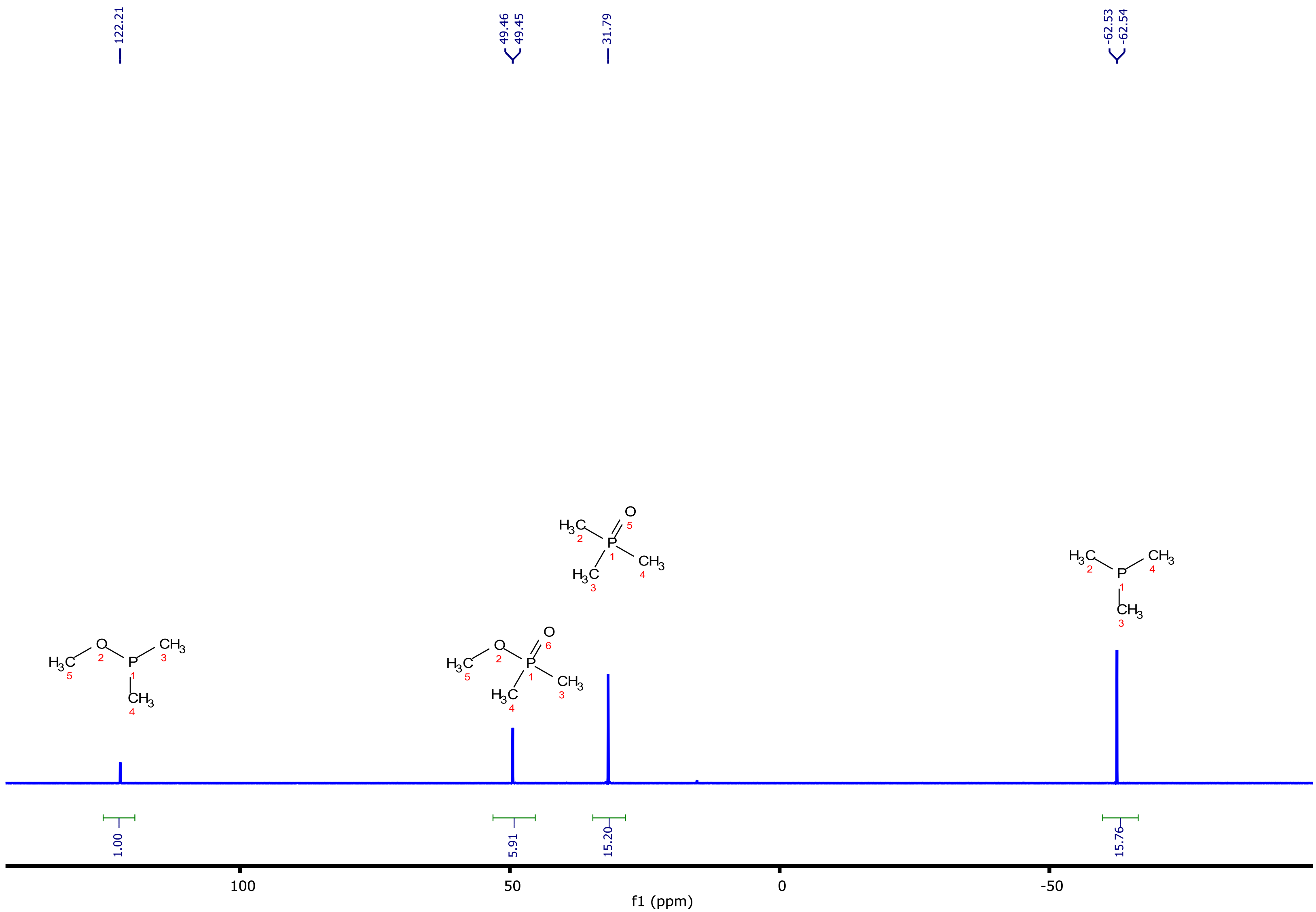

Figure S10. $^{31}P\{^{1}H\}$ spectrum of the reaction of PMe_3 with O_2 catalyzed with complex **1** in C_6D_6 in 100 mL Schlenk flask showing the pressence of three different oxidation products

6 References

(1) Vidovič, C.; Peschel, L. M.; Buchsteiner, M.; Belaj, F.; Mösch-Zanetti, N. C. Structural Mimics of Acetylene Hydratase: Tungsten Complexes Capable of Intramolecular Nucleophilic Attack on Acetylene. *Chem. Eur. J.* **2019**, *25* (63), 14267–14272.

(2) Ehweiner, M. A.; Wiedemaier, F.; Belaj, F.; Mösch-Zanetti, N. C. Oxygen Atom Transfer Reactivity of Molybdenum(VI) Complexes Employing Pyrimidine- and Pyridine-2-thiolate Ligands. *Inorg. Chem.* **2020**, *59* (19), 14577–14593.

(3) Ehweiner, M. A.; Wiedemaier, F.; Lajin, B.; Schachner, J. A.; Belaj, F.; Goessler, W.; Mösch-Zanetti, N. C. Nature-Inspired Homogeneous Catalytic Perchlorate Reduction Using Molybdenum Complexes. *ACS Catal.* **2021**, *11* (18), 11754–11761.

(4) Ćorović, M. Z.; Wiedemaier, F.; Belaj, F.; Mösch-Zanetti, N. C. Replacement of Molybdenum by Tungsten in a Biomimetic Complex Leads to an Increase in Oxygen Atom Transfer Catalytic Activity. *Inorg. Chem.* **2022**, *61* (31), 12415–12424.

(5) Kanishchev, O. S.; Dolbier, W. R. Synthesis and characterization of 2-pyridylsulfur pentafluorides. *Angew. Chem. Int. Ed.* **2015**, *54* (1), 280–284.

(6) Sheldrick, G. M. A short history of SHELX. *Acta Cryst.* **2008**, *A64*, 112–122.

(7) Sheldrick, G. M. Crystal structure refinement with SHELXL. *Acta Cryst.* **2015**, *71*, 3–8.

(8) Johnson, C. K. *ORTEP. Report ORNL-3794.*
